# Molecular architecture of mouse and human pancreatic zymogen granules: protein components and their copy numbers

**DOI:** 10.1007/s41048-018-0055-1

**Published:** 2018-04-26

**Authors:** Jin-sook Lee, Joseph A. Caruso, Garrett Hubbs, Patricia Schnepp, James Woods, Jingye Fang, Chunying Li, Kezhong Zhang, Paul M. Stemmer, Bhanu P. Jena, Xuequn Chen

**Affiliations:** 1 Department of Physiology, Wayne State University, Detroit, MI 48201 USA; 2 School of Medicine, Institute of Environmental Health Sciences, Wayne State University, Detroit, MI 48201 USA; 3 Department of Biochemistry and Molecular Biology, Wayne State University, Detroit, MI 48201 USA; 4 Center for Molecular Medicine and Genetics, Wayne State University, Detroit, MI 48201 USA

**Keywords:** Pancreatic zymogen granule, GeLC–MS/MS, LC-SRM, Absolute quantification, Copy numbers

## Abstract

A molecular model of pancreatic zymogen granule (ZG) is critical for understanding its functions. We have extensively characterized the composition and membrane topology of rat ZG proteins. In this study, we report the development of targeted proteomics approaches to quantify representative mouse and human ZG proteins using LC-SRM and heavy isotope-labeled synthetic peptides. The absolute quantities of mouse Rab3D and VAMP8 were determined as 1242 ± 218 and 2039 ± 151 (mean ± SEM) copies per ZG. The size distribution and the averaged diameter of ZGs 750 ± 23 nm (mean ± SEM) were determined by atomic force microscopy. The absolute quantification of Rab3D was then validated using semi-quantitative Western blotting with purified GST-Rab3D proteins as an internal standard. To extend our proteomics analysis to human pancreas, ZGs were purified using human acini obtained from pancreatic islet transplantation center. One hundred and eighty human ZG proteins were identified for the first time including both the membrane and the content proteins. Furthermore, the copy number per ZG of human Rab3D and VAMP8 were determined to be 1182 ± 45 and 485 ± 15 (mean ± SEM). The comprehensive proteomic analyses of mouse and human pancreatic ZGs have the potential to identify species-specific ZG proteins. The determination of protein copy numbers on pancreatic ZGs represents a significant advance towards building a quantitative molecular model of a prototypical secretory vesicle using targeted proteomics approaches. The identification of human ZG proteins lays a foundation for subsequent studies of altered ZG compositions and secretion in pancreatic diseases.

**Electronic supplementary material:**

The online version of this article (10.1007/s41048-018-0055-1) contains supplementary material, which is available to authorized users.

## Introduction

The acinar cells of the exocrine pancreas are the functional units of digestive enzyme synthesis, storage, and secretion. The zymogen granule (ZG) is the secretory organelle in the acinar cells responsible for transport, storage, and secretion of digestive enzymes and has long been a model for understanding secretory granule biogenesis and functions (Jamieson and Palade 1971a, b; Meldolesi *et al*. 1971; Messenger *et al*. 2014a; Williams 2001). The ZG content contains primarily the digestive enzymes and associated proteins which are the protein components of the pancreatic juice secreted into the duodenum (Chen *et al*. 2006, 2008; Doyle *et al*. 2012). The ZG membrane carries at least part of the molecular machinery responsible for digestive enzyme sorting, granule trafficking, and exocytosis (Chen *et al*. 2006, 2008; Gomez-Lazaro *et al*. 2010). Therefore, elucidating the ZG molecular architecture is critical for studying ZG functions. Defective ZG biogenesis results in various pancreatic diseases such as chronic and acute pancreatitis (Lerch and Gorelick 2013; Pandol *et al*. 2007; Sah *et al*. 2013; van Acker *et al*. 2006). The overall goal of our study is to build a quantitative, architectural model of the pancreatic ZG which will direct new hypotheses for subsequent functional analysis of this prototypic secretory granule. This model should ultimately comprise not only the complete protein components of the ZG, their membrane topologies, but also their absolute quantities and protein complexes they are associated with.

Towards this goal, we and others have carried out extensive characterizations of the components and membrane topology of rat ZGs (Chen *et al*. 2006, 2008; Rindler *et al*. 2007). Our new knowledge about novel ZG protein compositions has already led to a number of hypothesis-driven functional studies on individual ZG components (Chen *et al*. 2004; Faust *et al*. 2008; Hou *et al*. 2013; Sabbatini *et al*. 2008). A comprehensive model of the ZG requires the absolute quantity of each individual ZG protein as well as the stoichiometry among different ZG proteins. However, to date, this type of information has not yet been determined for any ZG protein. Here we apply an absolute quantification (AQUA) proteomics strategy using LC-SRM and isotope-labeled synthetic peptides to obtain absolute molar abundances for selected ZG proteins. Furthermore, by applying proteomics analysis to the purified human ZGs for the first time, we obtained the comprehensive constituents of human ZGs including both content and membrane proteins. The identification of human ZG-specific content and membrane proteins is expected to have a significant impact on translational studies to look for biomarker in pancreatic juice from cancer and pancreatitis patients.

## Results and discussion

### GeLC-SRM strategy for absolute quantification and copy number determination of representative ZG membrane proteins

**Fig. 1:**
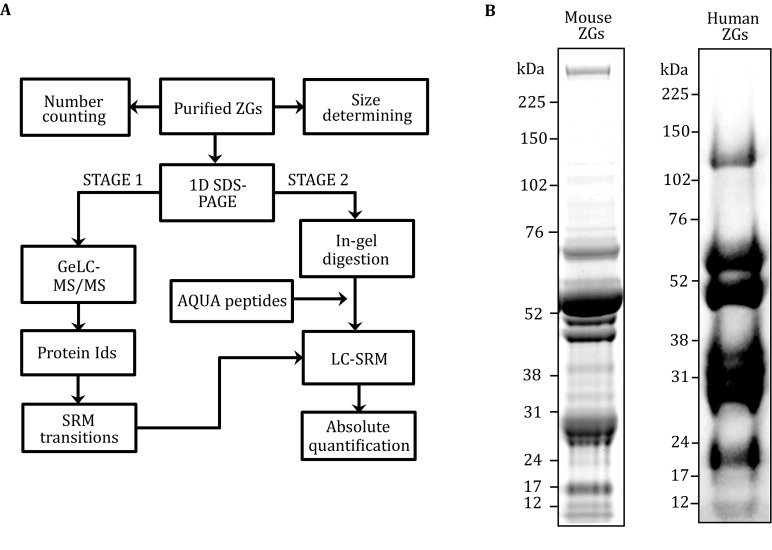
GeLC-SRM strategy for ZG copy number determination. **A** The workflow of AQUA strategy for determination of protein copy number per ZG. **B** Mouse (*left*) or human (*right*) ZG lysate was separated on 1D SDS-PAGE and stained with Coomassie Blue. Gel slices were excised from the entire lane for in-gel tryptic digestion and LC-MS/MS analysis

As described in Fig. 1A, ZGs were purified from mouse pancreas in each experiment. An aliquot of purified ZGs were used to determine their numbers and size distributions. The remaining ZGs were lyzed and separated on a SDS-PAGE gel in triplicate lanes (80 μg proteins per lane). ZG protein bands were stained with Coomassie Blue reagent, and the entire lane were excised in thirty gel slices for in-gel tryptic digestion (Fig. 1B). The tryptic peptides from each gel slice were then analyzed by LC–MS/MS. In a representative analysis of mouse pancreatic ZGs, 185 proteins were identified (supplemental Table S1) including a small number of potential contaminating proteins, *e.g.*, the 13 ribosomal subunit proteins. Two key technical modifications were made compared to previous studies (Chen *et al*. 2006, 2008). First, one mouse pancreas, instead of multiple rat pancreas, was used to obtain comparable numbers of protein identifications. Second, more importantly, whole ZG lysate was used in this study to avoid the steps to lyse ZGs and purify ZG membrane. This approach minimized the protein loss during sample processing, which is critical to the precise determination of protein abundance on ZGs. From these LC–MS/MS analyses, a Skyline mouse ZG protein spectral library was generated to facilitate the development of SRM transitions. In addition, the GeLC–MS/MS results also helped to define the position of a given protein on the gel (supplemental Fig. S1). This information allowed us to pool the tryptic digest from selected gel slices for the LC-SRM analysis. For the absolute quantification experiments, the heavy AQUA peptides were spiked in the tryptic digest after the in-gel digestion of the corresponding gel slices. Purified human ZGs were also analyzed following the same procedures (Fig. 1B).

### Determination of the optimal peptides and transitions for Rab3D and VAMP8 quantification

**Fig. 2:**
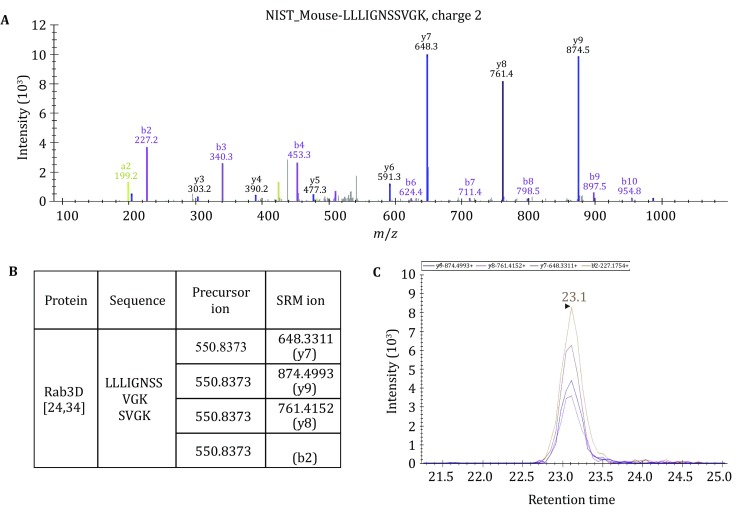
Development of SRM transitions for a unique Rab3D peptide. **A** MS/MS spectrum used to select the top ranked fragment ions for a unique Rab3D peptide. **B** Four SRM transitions developed for the peptide. **C** Extracted ion chromatograms of the four SRM transitions from LC-SRM analysis of combined in-gel tryptic digest of mouse ZG proteins around 30 kDa

Rab3D and VAMP8 are both present on ZG membrane and play critical roles in ZG trafficking and exocytosis (Cosen-Binker *et al*. 2008; Messenger *et al*. 2014b; Wang *et al*. 2007; Williams *et al*. 2009). As shown in supplemental Fig. S2, essentially all isolated ZGs were stained positive for Rab3D and VAMP8 in immunofluorescence. Therefore, we selected these two proteins as the representative ZG proteins to develop a LC-SRM procedure for their absolute quantification and copy number determination. Both Rab3D and VAMP8 are small proteins of relatively low abundance compared to the ZG content proteins (Chen *et al*. 2006). 1D SDS-PAGE allowed the separation of these two proteins from the majority of the abundant digestive enzymes of the ZG content and greatly enhanced the detection of unique tryptic peptides from these two proteins. In multiple GeLC–MS/MS experiments (supplemental Fig. S1), we could reproducibly detect the unique VAMP8 peptides from the bottom two gel bands (~12 kDa) and the unique Rab3D peptides from gel bands 5–9 (~30 kDa). Based on these LC–MS/MS results, a ZG spectral library was generated. This library together with the NIST (National Institute of Standards and Technology) mouse libraries of peptide tandem mass spectra were then used to develop the SRM transitions for Rab3D and VAMP8 using Skyline software (MacLean *et al*. 2010). Among all the peptides tested, two peptides were chosen based on their unique sequences and robust SRM signals. As shown in Fig. 2, peptide LLLIGNSSVGK was determined as an optimal sequence to quantify Rab3D. According to the BLAST analysis, this sequence is unique to Rab3D but not present in Rab3A, B, C, or in any other Rab proteins. The selected sequences do not have any cysteine, methionine, or internal tryptic cleavage site. This peptide is readily detectable from 1D gel separated mouse ZG lysate and can produce very high-quality MS/MS spectrum in different types of mass spectrometers (Fig. 2A). The top four most intense fragment ions, y7, y9, y8, and b2, were selected to develop the SRM transitions for this peptide (Fig. 2B). The LC-SRM ion chromatograms of all four transitions showed strong peaks at the same retention time (Fig. 2C) and there was essentially no significant signal above the noise level at any other retention time during the LC run. The same procedure was also applied to determine the unique VAMP8 peptide sequence, NLQSEVEGVK, and its SRM transitions using fragment ions of y7 (*m*/*z* 747.3883), y8 (*m*/*z* 875.4469), and b2 (*m*/*z* 228.1343) (data not shown). These results demonstrated that the unique peptides and corresponding SRM transitions were optimal to quantify Rab3D and VAMP8 protein abundance in mouse ZG lysate. Since both Rab3D and VAMP8 proteins are relatively small and sequence homology is high among isoforms, no other suitable peptides were found with both the strong SRM signals and the isoform specificity.

### Absolute quantification of Rab3D and VAMP8 in mouse ZGs using AQUA peptides

**Fig. 3:**
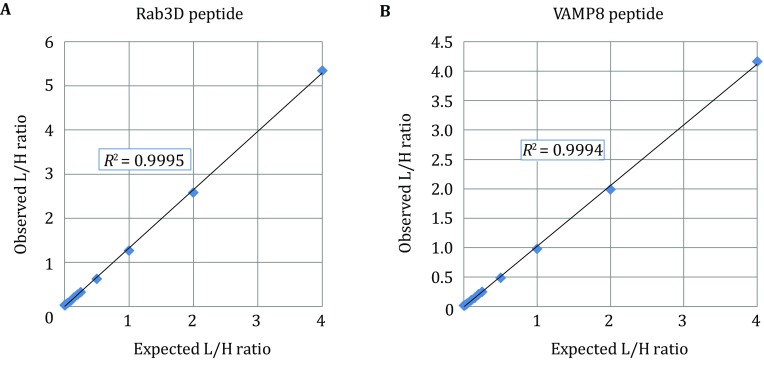
Calibration curves for the Rab3D and the VAMP8 AQUA peptides. Calibration curve samples were prepared by spiking increasing amounts of unlabeled synthetic Rab3D or VAMP8 target peptide (at 13 different concentrations from 0, 25 amol and then double each time until 16 fmol) into a complex peptide background made from the tryptic digest of *E. coli* total lysate. The isotope-labeled AQUA peptide standard for Rab3D or VAMP8 was added to each sample at 4 fmol. The LC-SRM signals were acquired for the light and heavy peptides in each sample. The ratios of the total peak areas, designated as observed L/H ratio, were calculated and plotted against the expected L/H ratios

**Fig. 4:**
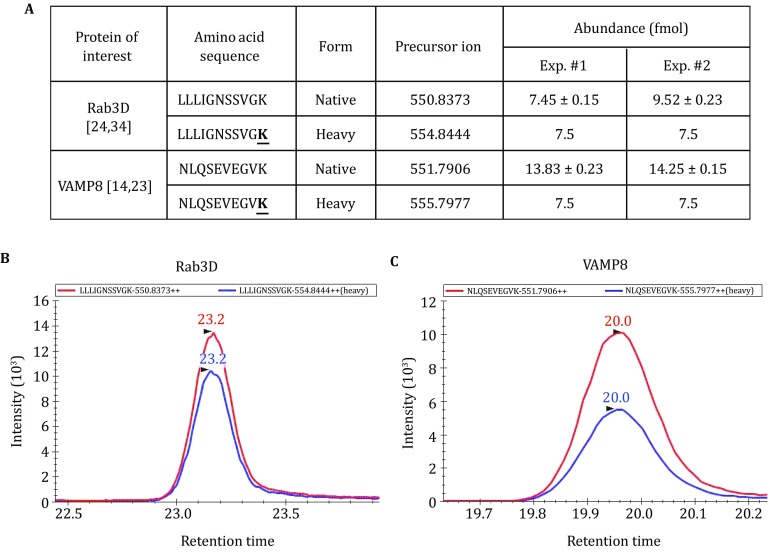
Absolute quantification of Rab3D and VAMP8 in mouse ZGs using AQUA peptides and LC-SRM analysis. **A** Unique AQUA peptides were used to quantify the abundance of Rab3D or VAMP8 in two separate mouse ZG preparations. The extracted ion chromatograms of the endogenous (*red*) and the heavy peptide (*blue*) for Rab3D (**B**) and VAMP8 (**C**) were shown from the experiment #2. The AUC ratio between the endogenous and the heavy peptide peaks was used to determine the protein absolute abundance (**A**)

For accurate quantitation of Rab3D and VAMP8, stable isotope-labeled target peptides (AQUA peptides) were spiked into ZG in-gel digests as internal standards. We first generated calibration curves for the two target peptides of Rab3D and VAMP8. Calibration curve samples were prepared by spiking increasing amounts of unlabeled synthetic peptides (at 13 different concentrations from 0, 25 amol and then double each time until 16 fmol) into a complex peptide background made from the tryptic digest of *E. coli* total lysate. The isotope-labeled AQUA peptide standards were added to each sample at a constant amount (4 fmol). The LC-SRM signals were acquired for the light (L) and heavy (H) peptides in each sample. The ratios of the total peak areas, designated as observed L/H ratio, were calculated and plotted (Fig. 3). These stable isotope dilution measurements showed low coefficients of variation (CV) across three replicate analyses (% CVs ranging from 0.29% to 6.77% at different concentrations for the Rab3D peptide and from 0.21% to 15.2% for the VAMP8 peptide). The linear correlation between the observed L/H ratios was obtained over three orders of magnitude from 0.025 − 16 fmol and *R*^2^ > 0.999 for both the Rab3D and VAMP8 peptides (Fig. 3). These results indicated that the target peptides were able to be quantified in a wide range without the interference from background peptides. We then determined the absolute quantities of Rab3D and VAMP8, respectively, using tryptic digests of the corresponding ZG gel slices (Fig. 4). For quantifying Rab3D, tryptic peptides from gel slices 5–9 were combined and 15 fmol of AQUA peptide, LLLIGNSSVGK, was spiked into the mixture. For VAMP8, tryptic peptides from gel slices 1 and 2 were combined with 15 fmol of AQUA peptide, NLQSEVEGVK. Duplicate samples from two separate ZG preparations (85 μg of mouse ZG lysate per lane) were run in parallel and GeLC-SRM was carried out in replicates. The area-under-the-curve (AUC) ratio between the endogenous and the heavy peptide peaks was used to determine the protein absolute abundance. The averaged absolute quantities and standard errors of the Rab3D peptide in two experiments were 0.44 ± 0.01 and 0.56 ± 0.01 fmol/μg ZG protein, respectively. The averaged absolute quantities and standard errors of the VAMP8 peptide were 0.81 ± 0.03 and 0.84 ± 0.02 fmol/μg protein, respectively.

To validate the absolute quantification by LC-SRM approach, we performed semi-quantitative Western blotting using purified GST-Rab3D as an internal standard. The purity of the GST-Rab3D recombinant protein was confirmed on a Coomassie Blue stained SDS-PAGE gel (data not shown). Next, we blotted ZG lysates from two separate preparations together with purified GST-Rab3D using anti-Rab3D antibody (supplemental Fig. S3) and estimated the average quantities of the endogenous Rab3D to be 0.45 and 0.41 fmol/μg ZG proteins, respectively. These results were in agreement with the results obtained from the LC-SRM analysis.

### Determination of the copy numbers per ZG and membrane densities of Rab3D and VAMP8

**Fig. 5:**
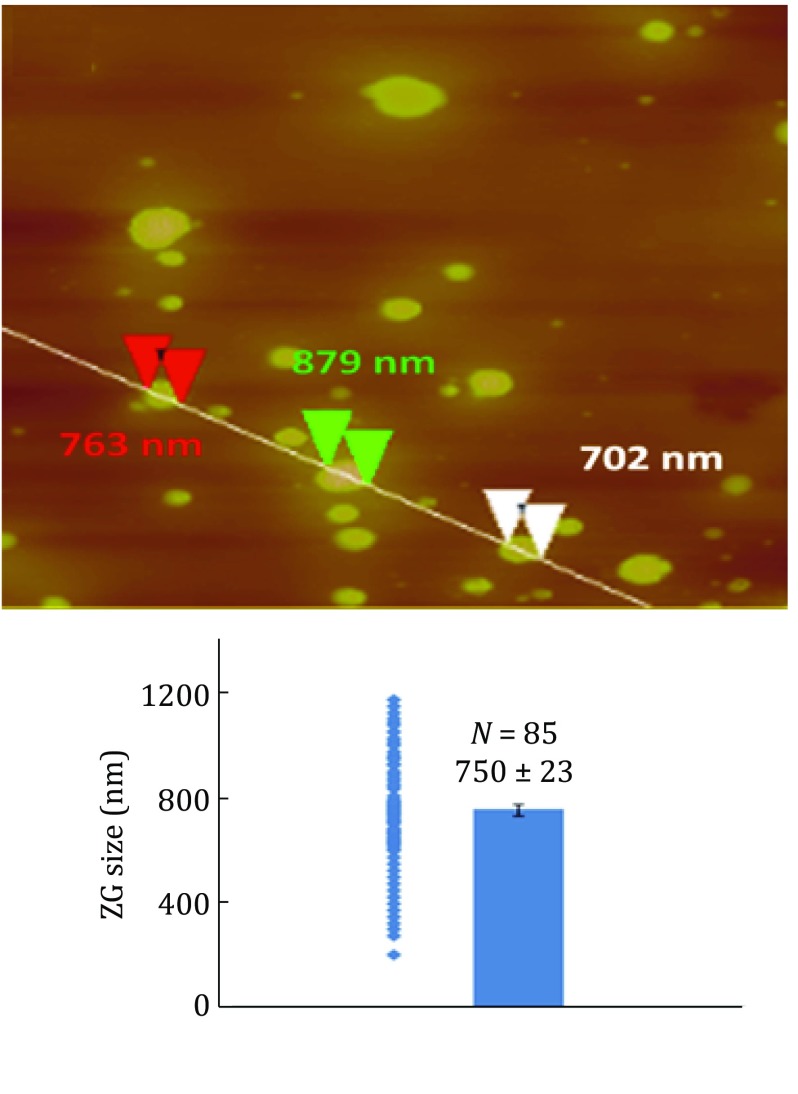
Determination of the size distribution of purified mouse ZGs by AFM. A representative AFM micrograph of purified ZGs and measurements of the diameters of three ZGs were marked with arrow heads (*top*). AFM measurements determined the size distribution and the average diameters of isolated mouse ZG (*bottom*, mean diameters ± SEM: 750 ± 23 nm, *N* = 85)

In order to convert the absolute quantifications of Rab3D and VAMP8 to their copy numbers per ZG, we took five aliquot of diluted ZG suspension and counted the numbers of ZGs per µl of suspension using hemocytometer. From five replicate counting, the averaged ZG number and standard error were 258,447 ± 18,090 per μg protein. With the numbers of ZGs measured, we were able to determine the copy numbers per ZG for Rab3D and VAMP8 based on the absolute quantification determined by LC-SRM and AQUA peptides. The averaged copy number/ZG of Rab3D was estimated to be 1242 ± 218 (mean ± SEM, 1024 in experiment 1 and 1460 in experiment 2). The averaged copy number/ZG of VAMP8 was 2039 ± 151 (mean ± SEM, 1887 in experiment 1 and 2190 in experiment 2). As shown in supplemental Fig. S2, the sizes of the isolated ZGs vary dramatically. To obtain the size distribution and the averaged size of ZGs, AFM morphometry was applied to the isolated ZG after fixation. A representative AFM image is shown in Fig. 5 top. In Fig. 5 bottom, the average diameter of isolated mouse ZGs was determined to be 750 ± 23 nm (mean ± SEM, *N* = 85). The size distribution and average diameter of ZG is consistent with previous reports using electron micrograph (Ermak and Rothman 1981; Liebow and Rothman 1973; Nadelhaft 1973). For an average sized ZG (750 nm in diameter), the densities of these two proteins, if evenly distributed on ZG membrane, were 702 molecules/μm^2^ for Rab3D and 1152 molecules/μm^2^ for VAMP8, respectively. As a comparison, it was estimated that Rab3A had 10 copies, and VAMP2 70 copies on a synaptic vesicle (an average diameter of 45.18 nm) with their corresponding membrane densities being 1572 and 11,003 molecules/μm^2^, respectively (Takamori *et al*. 2006). It is worth noting that some ZG proteins can be present on a subpopulation of ZGs or concentrated on specific domains of the ZG membrane. The average copy numbers and membrane densities determined here can serve as a starting point to further examine the uneven distributions of specific ZG proteins.

### Identification of human ZG proteins and absolute quantification of human Rab3D and VAMP8

To purify human ZGs, human acini from healthy donors were obtained from transplantation centers through Prodo Laboratories (Irvine, CA) and used as the starting material for homogenization. The subsequent steps were the same as in mouse ZG isolation. As shown in Fig. 1B, human ZG protein bands were stained with Coomassie Blue reagent and the entire lane were excised in thirty gel slices for in-gel tryptic digestion. In a GeLC–MS/MS analysis of human pancreatic ZGs, 180 proteins were identified including both membrane and content proteins (supplemental Table S2). A Skyline human ZG protein spectral library was generated to facilitate the development of SRM transitions. Previously, only the proteomics analyses of human pancreatic juice have been reported in normal and diseased subjects (Doyle *et al*. 2012; Gronborg *et al*. 2004; Paulo *et al*. 2013; Williams 2013). Our results represent the first effort to identify pancreatic ZG proteins from human pancreatic acini. These results have the potential to identify species-specific ZG proteins. In fact, several human ZG proteins, including ZA2G, REG3A and 3G, SYTL2, and O2AG1, have not been reported in our previous studies (Chen *et al*. 2006, 2008). We also highlighted the common proteins between our list and the reported proteins in normal human pancreatic juice (supplemental Table S2) (Doyle *et al*. 2012). Because pancreatic juice collected clinically sometimes contains the ZG content proteins as well as blood and cellular contaminants (Doyle *et al*. 2012), proteins found in both datasets represent potential bona fide ZG content proteins. Because the peptide sequences used to quantify mouse Rab3D and VAMP8 are conserved in human, we were able to obtain the absolute quantifications and determine the copy numbers per ZG for human Rab3D and VAMP8 using the same LC-SRM transitions and AQUA peptides. The averaged copy number per ZG of human Rab3D from two replicate analyses was 1182 ± 45 and that of VAMP8 was 485 ± 15 (mean ± SEM).

## Conclusions

In recent years, major advances have been made in the molecular mechanisms underlying membrane trafficking. However, quantitative information about the proteins or protein complexes involved has just begun to emerge. As the first time in any organelles, the copy numbers of over a dozen major constituents on synaptic vesicles were determined by semi-quantitative Western blotting (Takamori *et al*. 2006). However, this approach required specific antibodies and highly purified target proteins as standards, therefore, it is not suited for large-scale studies.

In this study, we performed comprehensive proteomic analyses of mouse and human pancreatic ZGs. These datasets allow the identification of species-specific ZG proteins. We then developed LC-SRM-based absolute quantification workflow using GeLC–MS and AQUA peptides. The numbers and size distribution of purified ZGs were also determined using atomic force and light microscopy. The determination of protein copy numbers and membrane densities on pancreatic ZGs represents a significant advance towards building a comprehensive molecular model, using quantitative proteomics approaches, for any given subcellular organelle. The identification of human ZG proteins also laid a foundation for subsequent studies of altered ZG compositions and secretion in pancreatic diseases.

## Materials and methods

### Reagents and materials

Percoll was purchased from MP Biomedicals (Santa Ana, CA, USA). Anti-Rab3D was a gift from Dr. M. McNiven and anti-VAMP8 antibody was from Synaptic Systems (Goettingen, Germany). Sequencing grade-modified trypsin was from Promega (Madison, WI, USA). Precolumn C18 reversed-phase cartridge and Acclain PepMap100 C18 reversed-phase analytical column were purchased from Dionex (Bannockburn, IL, USA). LC–MS grade water, acetonitrile (Optima), Halt protease inhibitors cocktail, and GelCode Blue Stain Reagent were purchased from Fisher Scientific (Pittsburgh, PA, USA). Other reagents were obtained from Sigma Chemical (St. Louis, MO, USA). All chemicals were of analytical grade and used as received. Heavy isotope-labeled synthetic peptides were purchased from Sigma and unlabeled synthetic peptides were from JPT Peptide Technologies (Berlin, Germany).

### Isolation of mouse and human zymogen granules

ZGs were purified as described in our early proteomics study (Chen *et al*. 2003, 2006, 2008). Briefly, pancreas was removed from an ICR mouse and homogenized in buffer containing 0.30 mol/L sucrose, 10 mmol/L MES (pH 6.0), 1 mmol/L EGTA, 0.1 mmol/L MgSO_4_, and 0.5 mmol/L phenylmethylsulfonyl fluoride. Homogenates were centrifuged at 300 *g* for 10 min at 4 °C, and then the supernatant was centrifuged again for another 10 min at 1800 *g* to generate a crude particulate fraction enriched in ZGs. This pellet was resuspended and mixed with equal volume of Percoll and centrifuged at 30,000 r/min (62,000 *g*) for 20 min in a Beckman ultracentrifuge using a Ti 70.1 rotor. The dense white ZG band was collected, pooled, and washed with homogenization buffer by recentrifugation. To purify human ZGs, human acini from healthy donors were obtained from islet transplantation centers through Prodo Laboratories (Irvine, CA). The human acini were shipped in a bottle of proprietary transport medium, which helps preserve functionality/viability. The cells were placed in a bottle via a shipping system that maintains a temperature range of 6–10 °C and shipped within 24 h of the isolation. The morphology and viability of human acini were evaluated. Majority (>95%) of acini were excluded from Trypan Blue staining. Unhealthy single acinar cells were separated from intact acini by gravity sedimentation in DMEM medium containing 4% BSA and soybean trypsin inhibitor. The acini pellet was used as the starting material for homogenization. The subsequent steps were the same as in mouse ZG isolation.

### GeLC–MS/MS analysis of mouse and human pancreatic ZGs

Purified ZGs were solubilized in lysis buffer and separated on 4%–12% 1D SDS-PAGE. The separated gel bands were stained with GelCode Blue Stain Reagent and the entire lane (~30 slices) were excised from the gel. In-gel protein digestion and nanoLC-MS/MS were performed as previously published (Chen *et al*. 2006; Lee *et al*. 2015). Briefly, the peptides were first separated on a reversed-phase C18 column with a 90 min gradient using the Dionex Ultimate HPLC system. MS and MS/MS spectra were then acquired on an Applied Biosystems QSTAR XL mass analyzer using information-dependent acquisition mode. Peaklists were then submitted to Mascot server V2.4.0 to search against the SwissProt_2014_06 database for Mus musculus (16,531 proteins) or homo sapiens (20,214 Proteins) with carbamidomethyl (C) as a fixed modification and oxidation (M), N-acetylation (protein N terminus) as variable modifications, 0 or 1 missed tryptic cleavage, 100 ppm mass tolerance for precursor ions, and 0.6 Da for the fragment ions. The Mascot search files were then imported to Scaffold for visualization and results output. To the proteins exported from Scaffold, a self-BLAST procedure was performed to further reduce protein redundancy. Tryptic peptides from purified human ZGs were also analyzed by LC–MS/MS on an EASY-nLC UHPLC system and Q Exactive™ Hybrid Quadrupole-Orbitrap mass spectrometer at the Wayne State University Proteomics core.

### Imaging analysis of isolated mouse pancreatic ZGs

As previously described (Chen *et al*. 2003, 2006), freshly purified ZGs were transferred onto microscope slides (Fisher Superfrost plus) and allowed to bind to slides for 10 min at room temperature. Then the PBS solution was replaced with 4% paraformaldehyde in PBS for 30 min at room temperature. After fixation, ZGs were blocked in 5% normal goat serum in PBS-0.2% Triton X-100 for 30 min at room temperature and then incubated with rabbit anti-Rab3D or rabbit anti-VAMP8 antibody diluted 1:200 in blocking solution for 1.5 h at room temperature. After washing with PBS, ZGs were incubated with Alexa Fluor 488 Chicken anti-rabbit IgG for 1 h at room temperature. Slides were viewed with Leica confocal microscope, and digitized images were processed using Photoshop software.

### Atomic force microscopy (AFM)

Isolated mouse ZGs were fixed in 2% paraformaldehyde/2% glutaraldehyde. Fixed ZGs in PBS (20 ul) were placed on mica surface, air-dried for 20 min, gently rinsed with distilled water to remove salt crystals, followed by N_2_ blow drying, and imaged in air tapping mode using the AFM (Nanoscope IIIa) from Digital Instruments (Santa Barbara, CA). The tip used was aluminum-coated silicon tips with a spring constant of 40 N/m, and an imaging force of <200 pN. Images were obtained at line frequencies of 1–2 Hz, with 256 lines per image, and 0.4 integral gain, 0.8 proportional gain. Topographical dimensions (height and lateral dimensions) of cellular structures were analyzed using “section analysis” in the software Nanoscope IIIa4.43r8, supplied by Digital Instruments.

### Targeted proteomics analysis and absolute quantification (AQUA) of representative ZG proteins

For selective reaction monitoring (SRM) experiments, the tryptic digest from selected gel slices was analyzed on a TSQ Vantage triple quadrupole mass spectrometer (Thermo Fisher Scientific, San Jose, CA) equipped with an Michrom nanoLC solvent delivery system, autosampler, and a nanospray source. The mobile phase was 0.1% formic acid in either HPLC grade water (Solvent A) or 100% acetonitrile (Solvent B). Peptides were separated on a Michrom Magic C18AQ 3 µ 200 A 0.1 × 150 cm column with a flow rate of 0.5 µl/min. A gradient of 2%–35% B was developed over 20 min. Transitions for each peptide were selected using the Skyline software package. Instrument parameters include Q2 gas 1.0 mTorr, scan width 0.004 Th, cycle time of 3 ms, and both Q1 and Q3 resolution FWHM (full width at half maximum) 0.7. Stable isotope-labeled synthetic peptides, LLLIGNSSVGK unique for Rab3D and NLQSEVEGVK unique for VAMP8, were used. The chemical purity of these peptides was more than 96% determined by LC-MS. The exact quantities of the synthetic peptides were determined by amino acid analysis. These AQUA peptides (15 fmol each) were spiked, as internal standards, into the corresponding tryptic digests for Rab3D or VAMP8, respectively. The intensity ratios between heavy peptide standard and endogenous peptide were calculated based on the MS peak area at FWHM of the chromatographic elution profile of each peptide pair using the Xcalibur software (Thermo Scientific, San Jose, CA). For the calibration curve studies, the non-heavy peptides with the same sequences were also synthesized. *E. coli* lysate was digested by trypsin and used as the background peptide mixture in the studies. Standard curves were developed by serial dilution of the light peptides against a constant concentration of heavy peptides in a background of *E. coli* tryptic digest. Data were analyzed using Skyline software (ver 2.5, MacCoss lab, Dept. of Genome Sciences, University of Washington), using AUC for each peak. The limit of detection was calculated by a signal-to-noise ratio of >3 and a CV of <25% for triplicate experiments. The limit of quantification was determined by identifying the next highest concentration on the standard curve with a signal-to-noise ratio of >10.

## Electronic supplementary material

Below is the link to the electronic supplementary material.

Supplementary material 1 (PDF 275 kb)

Supplementary material 2 (PDF 322 kb)

Supplementary material 3 (PDF 325 kb)

## References

[CR1] Chen X, Ernst SA, Williams JA (2003). Dominant negative Rab3D mutants reduce GTP-bound endogenous Rab3D in pancreatic acini. J Biol Chem.

[CR2] Chen X, Li C, Izumi T, Ernst SA, Andrews PC, Williams JA (2004). Rab27b localizes to zymogen granules and regulates pancreatic acinar exocytosis. Biochem Biophys Res Commun.

[CR3] Chen X, Walker AK, Strahler JR, Simon ES, Tomanicek-Volk SL, Nelson BB, Hurley MC, Ernst SA, Williams JA, Andrews PC (2006). Organellar proteomics: analysis of pancreatic zymogen granule membranes. Mol Cell Proteomics.

[CR4] Chen X, Ulintz PJ, Simon ES, Williams JA, Andrews PC (2008). Global topology analysis of pancreatic zymogen granule membrane proteins. Mol Cell Proteomics: MCP.

[CR5] Cosen-Binker LI, Binker MG, Wang CC, Hong W, Gaisano HY (2008). VAMP8 is the v-SNARE that mediates basolateral exocytosis in a mouse model of alcoholic pancreatitis. J Clin Invest.

[CR6] Doyle CJ, Yancey K, Pitt HA, Wang M, Bemis K, Yip-Schneider MT, Sherman ST, Lillemoe KD, Goggins MD, Schmidt CM (2012). The proteome of normal pancreatic juice. Pancreas.

[CR7] Ermak TH, Rothman SS (1981). Zymogen granules of pancreas decrease in size in response to feeding. Cell Tissue Res.

[CR8] Faust F, Gomez-Lazaro M, Borta H, Agricola B, Schrader M (2008). Rab8 is involved in zymogen granule formation in pancreatic acinar AR42 J cells. Traffic.

[CR9] Gomez-Lazaro M, Rinn C, Aroso M, Amado F, Schrader M (2010). Proteomic analysis of zymogen granules. Expert Rev Proteomics.

[CR10] Gronborg M, Bunkenborg J, Kristiansen TZ, Jensen ON, Yeo CJ, Hruban RH, Maitra A, Goggins MG, Pandey A (2004). Comprehensive proteomic analysis of human pancreatic juice. J Proteome Res.

[CR11] Hou Y, Chen X, Tolmachova T, Ernst SA, Williams JA (2013). EPI64B acts as a GTPase-activating protein for Rab27B in pancreatic acinar cells. J Biol Chem.

[CR12] Jamieson JD, Palade GE (1971). Condensing vacuole conversion and zymogen granule discharge in pancreatic exocrine cells: metabolic studies. J Cell Biol.

[CR13] Jamieson JD, Palade GE (1971). Synthesis, intracellular transport, and discharge of secretory proteins in stimulated pancreatic exocrine cells. J Cell Biol.

[CR14] Lee JS, Wu Y, Schnepp P, Fang J, Zhang X, Karnovsky A, Woods J, Stemmer PM, Liu M, Zhang K, Chen X (2015). Proteomics analysis of rough endoplasmic reticulum in pancreatic beta cells. Proteomics.

[CR15] Lerch MM, Gorelick FS (2013). Models of acute and chronic pancreatitis. Gastroenterology.

[CR16] Liebow C, Rothman SS (1973). Distribution of zymogen granule size. Am J Physiol.

[CR17] MacLean B, Tomazela DM, Shulman N, Chambers M, Finney GL, Frewen B, Kern R, Tabb DL, Liebler DC, MacCoss MJ (2010). Skyline: an open source document editor for creating and analyzing targeted proteomics experiments. Bioinformatics.

[CR18] Meldolesi J, Jamieson JD, Palade GE (1971). Composition of cellular membranes in the pancreas of the guinea pig. I. Isolation of membrane fractions. J Cell Biol.

[CR19] Messenger SW, Falkowski MA, Groblewski GE (2014). Ca(2)(+)-regulated secretory granule exocytosis in pancreatic and parotid acinar cells. Cell Calcium.

[CR20] Messenger SW, Falkowski MA, Thomas DD, Jones EK, Hong W, Gaisano HY, Boulis NM, Groblewski GE (2014). Vesicle associated membrane protein 8 (VAMP8)-mediated zymogen granule exocytosis is dependent on endosomal trafficking via the constitutive-like secretory pathway. J Biol Chem.

[CR21] Nadelhaft I (1973). Measurement of the size distribution of zymogen granules from rat pancreas. Biophys J.

[CR22] Pandol SJ, Saluja AK, Imrie CW, Banks PA (2007). Acute pancreatitis: bench to the bedside. Gastroenterology.

[CR23] Paulo JA, Kadiyala V, Brizard S, Banks PA, Steen H, Conwell DL (2013). Post-translational modifications of pancreatic fluid proteins collected via the endoscopic pancreatic function test (ePFT). J Proteomics.

[CR24] Rindler MJ, Xu CF, Gumper I, Smith NN, Neubert TA (2007). Proteomic analysis of pancreatic zymogen granules: identification of new granule proteins. J Proteome Res.

[CR25] Sabbatini ME, Chen X, Ernst SA, Williams JA (2008). Rap1 activation plays a regulatory role in pancreatic amylase secretion. J Biol Chem.

[CR26] Sah RP, Dawra RK, Saluja AK (2013). New insights into the pathogenesis of pancreatitis. Curr Opin Gastroen.

[CR27] Takamori S, Holt M, Stenius K, Lemke EA, Gronborg M, Riedel D, Urlaub H, Schenck S, Brugger B, Ringler P, Müller SA, Rammner B, Gräter F, Hub JS, De Groot BL, Mieskes G, Moriyama Y, Klingauf J, Grubmüller H, Heuser J, Wieland F, Jahn R (2006). Molecular anatomy of a trafficking organelle. Cell.

[CR28] van Acker GJ, Perides G, Steer ML (2006). Co-localization hypothesis: a mechanism for the intrapancreatic activation of digestive enzymes during the early phases of acute pancreatitis. World J Gastroenterol.

[CR29] Wang CC, Shi H, Guo K, Ng CP, Li J, Gan BQ, Chien Liew H, Leinonen J, Rajaniemi H, Zhou ZH, Zeng Q, Hong W (2007). VAMP8/endobrevin as a general vesicular SNARE for regulated exocytosis of the exocrine system. Mol Biol Cell.

[CR30] Williams JA (2001). Intracellular signaling mechanisms activated by cholecystokinin-regulating synthesis and secretion of digestive enzymes in pancreatic acinar cells. Annu Rev Physiol.

[CR31] Williams JA (2013). Proteomics as a systems approach to pancreatitis. Pancreas.

[CR32] Williams JA, Chen X, Sabbatini ME (2009). Small G proteins as key regulators of pancreatic digestive enzyme secretion. Am J Physiol Endocrinol Metab.

