# Supplementary material for: Molecular architecture of mouse and human pancreatic zymogen granules: protein components and their copy numbers
Source: Biophys Rep. 2018 Apr 26;4(2):94–103. doi: 10.1007/s41048-018-0055-1 (PMC5937866; doi:10.1007/s41048-018-0055-1)
Supplement: Supplementary file 1 — Supplementary material 1 (PDF 275 kb) [file 41048_2018_55_MOESM1_ESM.pdf]

Supplemental figures

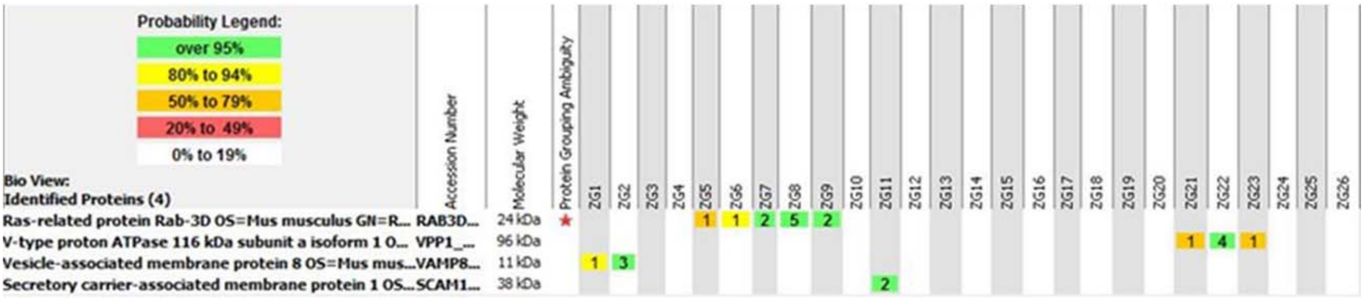

Fig. S1 Detection of representative ZG proteins in restricted regions of a 1D SDS-PAGE gel. The number of unique peptides detected in each gel slice for Rab3D, VAMP8, V-ATPase a1 and SCAMP1 were illustrated across all the gel slices in the entire lane

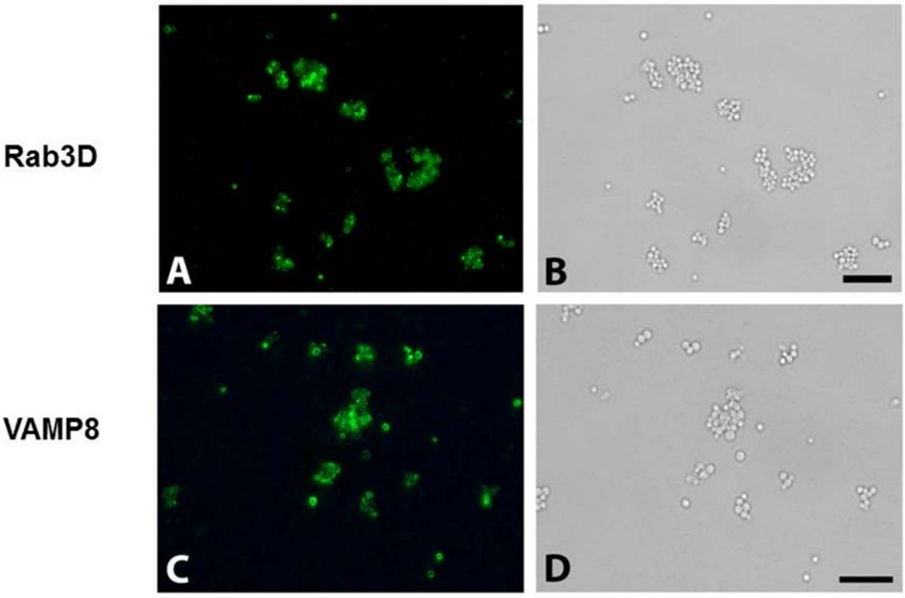

Fig. S2 Immunolocalization of ZG proteins on purified mouse ZGs. Immunocytochemical detection of Rab3D (**A**) and VAMP8 (**C**) from isolated mouse ZGs using confocal microscopy. ZG fluorescence images are paired with the corresponding bright field images (**B**) and (**D**). Scale bar = 5 µm

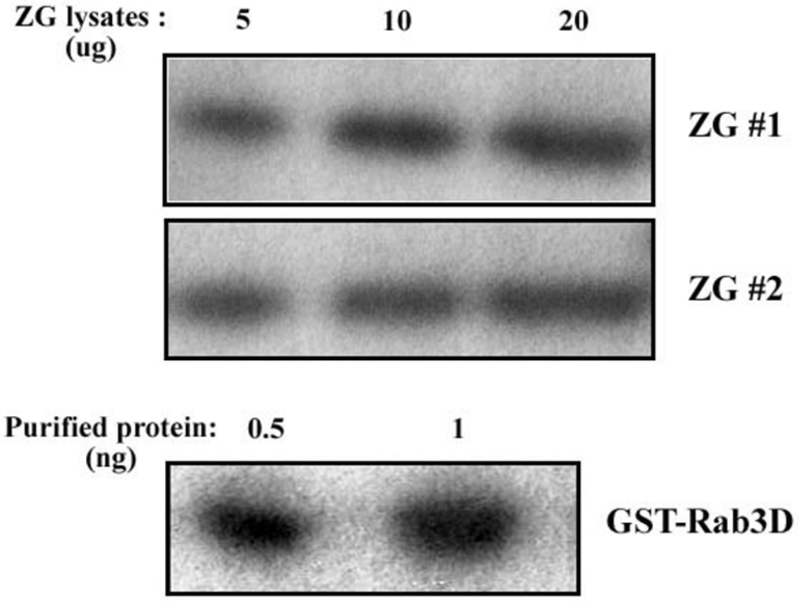

Fig. S3 Quantification of Rab3D using WB and GST-Rab3D as the internal standard. Increased amounts of mouse ZG lysates from two separated preparations were separated, together with known amounts of the purified GST-Rab3D protein, on a 1D SDS-PAGE. The samples were then Western blotted with anti-Rab3D antibody. The abundances of Rab3D in mouse ZGs were determined by densitometry using GST-Rab3D signals as the internal standard
