# Supplementary material for: Molecular architecture of mouse and human pancreatic zymogen granules: protein components and their copy numbers
Source: Biophys Rep. 2018 Apr 26;4(2):94–103. doi: 10.1007/s41048-018-0055-1 (PMC5937866; doi:10.1007/s41048-018-0055-1)
Supplement: Supplementary file 2 — Supplementary material 2 (PDF 322 kb) [file 41048_2018_55_MOESM2_ESM.pdf]

Supplemental Table 1: Proteins identified from isolated mouse ZGs. Purified mouse ZGs (~ 80 ug proteins) were analyzed by 1D SDS-PAGE coupled to LC–MS/MS on QSTAR XL. Protein identifications are reported with protein and peptide confidence ≥90%. The protein names, accesion numbers in UniProt, molecular weights and numbers of peptides identified are listed in separated columns.

Identified Proteins	Accession Number	Molecular Weight	# of Spectra
Pancreatic alpha-amylase	AMYP_MOUSE	57 kDa	387
Pancreatic triacylglycerol lipase	LIPP_MOUSE	51 kDa	146
Bile salt-activated lipase	CEL_MOUSE	66 kDa	142
Chymotrypsin-like elastase family member 2A	CEL2A_MOUSE	29 kDa	130
Pancreatic lipase-related protein 1	LIPR1_MOUSE	53 kDa	127
Chymotrypsinogen B	CTRB1_MOUSE	28 kDa	122
Carboxypeptidase A1	CBPA1_MOUSE	47 kDa	96
Deleted in malignant brain tumors 1 protein	DMBT1_MOUSE	227 kDa	73
Protein disulfide-isomerase	PDIA1_MOUSE	57 kDa	56
Zymogen granule membrane protein 16	ZG16_MOUSE	18 kDa	55
Anionic trypsin-2	TRY2_MOUSE	26 kDa	46
Kallikrein-1	KLK1_MOUSE	29 kDa	40
78 kDa glucose-regulated protein	GRP78_MOUSE	72 kDa	39
Desmoplakin	DESP_MOUSE	333 kDa	34
Pancreatic secretory granule membrane major glycoprotein GP2	GP2_MOUSE	59 kDa	33
CUB and zona pellucida-like domain-containing protein 1	CUZD1_MOUSE	68 kDa	32
Serum albumin	ALBU_MOUSE	69 kDa	31
Lithostathine-1	LIT1_MOUSE	19 kDa	28
ATP synthase subunit beta, mitochondrial	ATPB_MOUSE	56 kDa	27
Pancreatic lipase-related protein 2	LIPR2_MOUSE	53 kDa	25
Clusterin	CLUS_MOUSE	52 kDa	25
Pyruvate carboxylase, mitochondrial	PYC_MOUSE	130 kDa	24
Carboxypeptidase A2	CBPA2_MOUSE	47 kDa	24
Ribonuclease pancreatic	RNAS1_MOUSE	17 kDa	24
Colipase	COL_MOUSE	12 kDa	24
Junction plakoglobin	PLAK_MOUSE	82 kDa	23

Hemoglobin subunit beta-1	HBB1_MOUSE	16 kDa	23
Chymotrypsin-C	CTRC_MOUSE	29 kDa	22
Serpin I2	SPI2_MOUSE	46 kDa	21
Protein disulfide-isomerase A2	PDIA2_MOUSE	58 kDa	20
Gamma-glutamyltranspeptidase 1	GGT1_MOUSE	62 kDa	18
Syncollin	SYCN_MOUSE	15 kDa	17
Synaptic vesicle membrane protein VAT-1 homolog	VAT1_MOUSE	43 kDa	16
Endoplasmin	ENPL_MOUSE	92 kDa	15
Guanine nucleotide-binding protein subunit beta-2-like 1	GBLP_MOUSE	35 kDa	15
Delta-1-pyrroline-5-carboxylate synthase	P5CS_MOUSE	87 kDa	15
40S ribosomal protein SA	RSSA_MOUSE	33 kDa	15
Dolichyl-diphosphooligaccharide--protein glycosyltransferase subunit	RPN1_MOUSE	69 kDa	14
Protein disulfide-isomerase A6	PDIA6_MOUSE	48 kDa	14
ADP/ATP translocase 2	ADT2_MOUSE	33 kDa	14
60S acidic ribosomal protein P0	RLA0_MOUSE	34 kDa	13
Isobutyryl-CoA dehydrogenase, mitochondrial	ACAD8_MOUSE	45 kDa	12
Elongation factor 1-alpha 1	EF1A1_MOUSE	50 kDa	12
Electron transfer flavoprotein subunit beta	ETFB_MOUSE	28 kDa	12
Staphylococcal nuclease domain-containing protein 1	SND1_MOUSE	102 kDa	11
Nucleobindin-2	NUCB2_MOUSE	50 kDa	11
Hemoglobin subunit alpha	HBA_MOUSE	15 kDa	11
Voltage-dependent anion-selective channel protein 2	VDAC2_MOUSE	32 kDa	11
Ras-related protein Rab-3D	RAB3D_MOUSE	24 kDa	11
Peptidyl-prolyl cis-trans isomerase B	PPIB_MOUSE	24 kDa	10
UPF0577 protein KIAA1324	K1324_MOUSE	111 kDa	10
Elongation factor 2	EF2_MOUSE	95 kDa	9
Leucine-rich repeat-containing protein 59	LRC59_MOUSE	35 kDa	9
Cystatin-C	CYTC_MOUSE	16 kDa	9
Protein disulfide-isomerase A3	PDIA3_MOUSE	57 kDa	8
Lithostathine-2	LIT2_MOUSE	19 kDa	8
Protein transport protein Sec61 subunit alpha isoform 1	S61A1_MOUSE	52 kDa	8
V-type proton ATPase 116 kDa subunit a isoform 1	VPP1_MOUSE	96 kDa	7

Voltage-dependent anion-selective channel protein 1	VDAC1_MOUSE	32 kDa	7
Trans-2-enoyl-CoA reductase, mitochondrial	MECR_MOUSE	40 kDa	7
Prohibitin-2	PHB2_MOUSE	33 kDa	7
Signal recognition particle receptor subunit alpha	SRPR_MOUSE	70 kDa	7
Protein disulfide-isomerase A4	PDIA4_MOUSE	72 kDa	7
Sideroflexin-1	SFXN1_MOUSE	36 kDa	7
Carboxypeptidase D	CBPD_MOUSE	152 kDa	6
Vigilin	VIGLN_MOUSE	142 kDa	6
Sulfhydryl oxidase 1	QSOX1_MOUSE	83 kDa	6
Translocon-associated protein subunit delta	SSRD_MOUSE	19 kDa	6
40S ribosomal protein S3	RS3_MOUSE	27 kDa	6
V-type proton ATPase subunit d 1	VA0D1_MOUSE	40 kDa	6
Lysosome-associated membrane glycoprotein 2	LAMP2_MOUSE	46 kDa	6
40S ribosomal protein S12	RS12_MOUSE	15 kDa	6
Hemoglobin subunit beta-2	HBB2_MOUSE	16 kDa	6
Thiosulfate sulfurtransferase	THTR_MOUSE	33 kDa	5
17-beta-hydroxysteroid dehydrogenase 13	DHB13_MOUSE	34 kDa	5
Cathepsin D	CATD_MOUSE	45 kDa	5
Solute carrier family 25 member 35	S2535_MOUSE	33 kDa	5
Alpha-soluble NSF attachment protein	SNAA_MOUSE	33 kDa	5
Cytochrome c oxidase subunit 2	COX2_MOUSE	26 kDa	5
60S ribosomal protein L12	RL12_MOUSE	18 kDa	5
Annexin A2	ANXA2_MOUSE	39 kDa	5
Neutral alpha-glucosidase AB	GANAB_MOUSE	107 kDa	5
Transmembrane protein 63A	TM63A_MOUSE	92 kDa	5
Peptidyl-prolyl cis-trans isomerase FKBP11	FKB11_MOUSE	22 kDa	5
ERO1-like protein beta	ERO1B_MOUSE	54 kDa	5
Actin, cytoplasmic 1	ACTB_MOUSE	42 kDa	4
Ras-related C3 botulinum toxin substrate 1	RAC1_MOUSE	21 kDa	4
Carbohydrate kinase domain-containing protein	CARKD_MOUSE	37 kDa	4
AP-1 complex subunit gamma-1	AP1G1_MOUSE	91 kDa	4
Vesicle-associated membrane protein 3	VAMP3_MOUSE	11 kDa	4

Guanine nucleotide-binding protein G(I)/G(S)/G(T) subunit beta-2	GBB2_MOUSE	37 kDa	4
Alpha-aminoadipic semialdehyde dehydrogenase	AL7A1_MOUSE	59 kDa	4
Sarcoplasmic/endoplasmic reticulum calcium ATPase 2	AT2A2_MOUSE	115 kDa	4
Heat shock cognate 71 kDa protein	HSP7C_MOUSE	71 kDa	4
Vesicle-associated membrane protein 8	VAMP8_MOUSE	11 kDa	4
Nucleoside diphosphate kinase B	NDKB_MOUSE	17 kDa	4
Dolichyl-diphosphooligaccharide--protein glycosyltransferase 48 kDa s	OST48_MOUSE	49 kDa	4
40S ribosomal protein S25	RS25_MOUSE	14 kDa	4
Voltage-dependent anion-selective channel protein 3	VDAC3_MOUSE	31 kDa	4
Transmembrane protein 214	TM214_MOUSE	76 kDa	4
4F2 cell-surface antigen heavy chain	4F2_MOUSE	58 kDa	4
Hypoxia up-regulated protein 1	HYOU1_MOUSE	111 kDa	4
Phospholipase A2	PA21B_MOUSE	16 kDa	4
Endoplasmic reticulum resident protein 27	ERP27_MOUSE	31 kDa	4
Alpha-amylase 1	AMY1_MOUSE	58 kDa	3
Alpha-enolase	ENOA_MOUSE	47 kDa	3
Plakophilin-1	PKP1_MOUSE	81 kDa	3
Galectin-12	LEG12_MOUSE	35 kDa	3
Gamma-glutamyl hydrolase	GGH_MOUSE	35 kDa	3
Signal peptidase complex catalytic subunit SEC11C	SC11C_MOUSE	22 kDa	3
Pyrroline-5-carboxylate reductase 2	P5CR2_MOUSE	34 kDa	3
MOSC domain-containing protein 2, mitochondrial	MOSC2_MOUSE	38 kDa	3
40S ribosomal protein S3a	RS3A_MOUSE	30 kDa	3
Trefoil factor 2	TFF2_MOUSE	14 kDa	3
Citrate synthase, mitochondrial	CISY_MOUSE	52 kDa	3
Ras-related protein Rab-1A	RAB1A_MOUSE	23 kDa	3
60S ribosomal protein L22	RL22_MOUSE	15 kDa	3
Cytochrome c, somatic	CYC_MOUSE	12 kDa	3
Nodal modulator 1	NOMO1_MOUSE	133 kDa	3
60S ribosomal protein L5	RL5_MOUSE	34 kDa	3
Cytochrome c oxidase subunit 6B1	CX6B1_MOUSE	10 kDa	3
Prohibitin	PHB_MOUSE	30 kDa	3

Transforming protein RhoA	RHOA_MOUSE	22 kDa	3
Protein canopy homolog 2	CNPY2_MOUSE	21 kDa	3
Cytochrome b5 type B	CYB5B_MOUSE	16 kDa	3
Dolichyl-diphosphooligosaccharide--protein glycosyltransferase subur	DAD1_MOUSE	12 kDa	3
40S ribosomal protein S4, X isoform	RS4X_MOUSE	30 kDa	3
Synaptophysin-like protein 1	SYPL1_MOUSE	29 kDa	3
NADH-cytochrome b5 reductase 3	NB5R3_MOUSE	34 kDa	3
C-1-tetrahydrofolate synthase, cytoplasmic	C1TC_MOUSE	101 kDa	3
CDGSH iron-sulfur domain-containing protein 1	CISD1_MOUSE	12 kDa	3
NADH dehydrogenase [ubiquinone] 1 alpha subcomplex subunit 4	NDUA4_MOUSE	9 kDa	3
NADH dehydrogenase [ubiquinone] 1 alpha subcomplex subunit 12	NDUAC_MOUSE	17 kDa	3
Cytochrome b-c1 complex subunit 7	QCR7_MOUSE	14 kDa	3
60S ribosomal protein L23	RL23_MOUSE	15 kDa	3
Dolichyl-diphosphooligosaccharide--protein glycosyltransferase subur	RPN2_MOUSE	69 kDa	3
40S ribosomal protein S14	RS14_MOUSE	16 kDa	3
Vacuolar protein sorting-associated protein 45	VPS45_MOUSE	65 kDa	3
Zinc-binding alcohol dehydrogenase domain-containing protein 2	ZADH2_MOUSE	41 kDa	3
Ras-related protein Rap-1A	RAP1A_MOUSE	21 kDa	3
Cytoplasmic dynein 1 light intermediate chain 1	DC1L1_MOUSE	57 kDa	3
Peroxiredoxin-4	PRDX4_MOUSE	31 kDa	3
Protein Daple	DAPLE_MOUSE	227 kDa	2
60S acidic ribosomal protein P2	RLA2_MOUSE	12 kDa	2
NADH dehydrogenase [ubiquinone] 1 beta subcomplex subunit 10	NDUBA_MOUSE	21 kDa	2
40S ribosomal protein S21	RS21_MOUSE	9 kDa	2
Interferon-induced transmembrane protein 3	IFM3_MOUSE	15 kDa	2
Brain protein 44	BR44_MOUSE	14 kDa	2
Ubiquinone biosynthesis monooxygenase COQ6	COQ6_MOUSE	51 kDa	2
UDP-glucose:glycoprotein glucosyltransferase 1	UGGG1_MOUSE	176 kDa	2
Sideroflexin-3	SFXN3_MOUSE	35 kDa	2
Mesencephalic astrocyte-derived neurotrophic factor	MANF_MOUSE	20 kDa	2
40S ribosomalprotein S18	RS18_MOUSE	18 kDa	2
40S ribosomal protein S10	RS10_MOUSE	19 kDa	2

AP-1 complex subunit beta-1	AP1B1_MOUSE	104 kDa	2
Kallikrein 1-related peptidase b5	K1KB5_MOUSE	29 kDa	2
Dolichyl-phosphate beta-glucosyltransferase	ALG5_MOUSE	37 kDa	2
Aminopeptidase N	AMPN_MOUSE	110 kDa	2
40S ribosomal protein S2	RS2_MOUSE	31 kDa	2
Actin-related protein 2/3 complex subunit 4	ARPC4_MOUSE	20 kDa	2
Peroxisomal trans-2-enoyl-CoA reductase	PECR_MOUSE	32 kDa	2
CDGSH iron-sulfur domain-containing protein 2	CISD2_MOUSE	15 kDa	2
ADP-ribosylation factor 1	ARF1_MOUSE	21 kDa	2
ADP-ribosylation factor 6	ARF6_MOUSE	20 kDa	2
Fumarylacetoacetate hydrolase domain-containing protein 2A	FAHD2_MOUSE	35 kDa	2
Glucagon	GLUC_MOUSE	21 kDa	2
Pyruvate kinase isozymes M1/M2	KPYM_MOUSE	58 kDa	2
Alpha-mannosidase 2	MA2A1_MOUSE	132 kDa	2
Malectin	MLEC_MOUSE	32 kDa	2
Omega-amidase NIT2	NIT2_MOUSE	31 kDa	2
Vesicle-fusing ATPase	NSF_MOUSE	83 kDa	2
Polymerase delta-interacting protein 2	PDIP2_MOUSE	42 kDa	2
Palmitoyl-protein thioesterase 1	PPT1_MOUSE	34 kDa	2
Ras-related protein Rab-27A	RB27A_MOUSE	25 kDa	2
Rho GTPase-activating protein 1	RHG01_MOUSE	50 kDa	2
60S ribosomal protein L9	RL9_MOUSE	22 kDa	2
40S ribosomal protein S11	RS11_MOUSE	18 kDa	2
40S ribosomal protein S15a	RS15A_MOUSE	15 kDa	2
40S ribosomal protein S19	RS19_MOUSE	16 kDa	2
Protein transport protein Sec61 subunit beta	SC61B_MOUSE	10 kDa	2
Secretory carrier-associated membrane protein 1	SCAM1_MOUSE	38 kDa	2
Signal recognition particle receptor subunit beta	SRPRB_MOUSE	30 kDa	2
TraB domain-containing protein	TRABD_MOUSE	42 kDa	2
Ribonuclease UK114	UK114_MOUSE	14 kDa	2
Signal peptidase complex subunit 2	SPCS2_MOUSE	25 kDa	2
