# Supplementary material for: Molecular architecture of mouse and human pancreatic zymogen granules: protein components and their copy numbers
Source: Biophys Rep. 2018 Apr 26;4(2):94–103. doi: 10.1007/s41048-018-0055-1 (PMC5937866; doi:10.1007/s41048-018-0055-1)
Supplement: Supplementary file 3 — Supplementary material 3 (PDF 325 kb) [file 41048_2018_55_MOESM3_ESM.pdf]

Supplemental Table 2: Proteins identified from isolated human ZGs. Purified human ZGs (~ 50 ug proteins) were analyzed by 1D SDS-PAGE coupled to LC–MS/MS on QSTAR XL. Protein identifications are reported with protein and peptide confidence ≥90%. The protein names, accesion numbers in UniProt, molecular weights and numbers of peptides identified are listed in separated columns. In addition, proteins also reported in normal human pancreatic juice are marked as "*" in a column called "Found in pancreatic juice".

Identified Proteins	Accession Number	MW	# of spectra	Found in pancreatic juice
Carboxypeptidase B	CBPB1_HUMAN	47 kDa	270	*
Carboxypeptidase A1	CBPA1_HUMAN	47 kDa	243	*
Bile salt-activated lipase	CEL_HUMAN	79 kDa	213	*
Pancreatic triacylglycerol lipase	LIPP_HUMAN	51 kDa	176	*
Carboxypeptidase A2	CBPA2_HUMAN	47 kDa	118	*
Pancreatic alpha-amylase	AMYP_HUMAN	58 kDa	116	*
Chymotrypsin-like elastase family member 3A	CEL3A_HUMAN	29 kDa	114	*
Alpha-amylase 2B	AMY2B_HUMAN	58 kDa	107	*
Pancreatic lipase-related protein 2	LIPR2_HUMAN	52 kDa	91	*
Trypsin-2	TRY2_HUMAN	26 kDa	88	*
Chymotrypsinogen B2	CTRB2_HUMAN	28 kDa	85	*
Chymotrypsin-like elastase family member 3B	CEL3B_HUMAN	29 kDa	85	*
Chymotrypsin-C	CTRC_HUMAN	29 kDa	82	*
Trypsin-1	TRY1_HUMAN	27 kDa	80	*
Lithostathine-1-alpha	REG1A_HUMAN	19 kDa	72	*
Pancreatic secretory granule membrane major glycoprotein GP2	GP2_HUMAN	59 kDa	68	*
Lithostathine-1-beta	REG1B_HUMAN	19 kDa	68	*
Trypsin-3	TRY3_HUMAN	33 kDa	57	
Chymotrypsin-like elastase family member 2A	CEL2A_HUMAN	29 kDa	45	*
Inactive pancreatic lipase-related protein 1	LIPR1_HUMAN	52 kDa	35	*
Chymotrypsin-like elastase family member 2B	CEL2B_HUMAN	29 kDa	28	
CUB and zona pellucida-like domain-containing protein 1	CUZD1_HUMAN	68 kDa	27	*
Phospholipase A2	PA21B_HUMAN	16 kDa	23	*
Alpha-enolase	ENOA_HUMAN	47 kDa	21	*
Chymotrypsin-like protease CTRL-1	CTRL_HUMAN	28 kDa	19	

Kallikrein-1	KLK1_HUMAN	29 kDa	18	*
Complement C4-A	CO4A_HUMAN	193 kDa	14	*
Colipase	COL_HUMAN	12 kDa	14	*
Alpha-1-antichymotrypsin	AACT_HUMAN	48 kDa	12	
Serpin I2	SPI2_HUMAN	46 kDa	12	
Tubulin beta-2A chain	TBB2A_HUMAN	50 kDa	12	
CD63 antigen	CD63_HUMAN	26 kDa	9	
Clusterin	CLUS_HUMAN	52 kDa	9	
Dermcidin	DCD_HUMAN	11 kDa	9	
78 kDa glucose-regulated protein	GRP78_HUMAN	72 kDa	8	
Unconventional myosin-Vc	MYO5C_HUMAN	203 kDa	8	
Plastin-2	PLSL_HUMAN	70 kDa	8	
Peptidyl-prolyl cis-trans isomerase A	PPIA_HUMAN	18 kDa	8	*
Cardiotrophin-like cytokine factor 1	CLCF1_HUMAN	25 kDa	7	
Protein disulfide-isomerase A4	PDIA4_HUMAN	73 kDa	7	
Regenerating islet-derived protein 3-gamma	REG3G_HUMAN	19 kDa	7	
Protein disulfide-isomerase A2	PDIA2_HUMAN	58 kDa	7	
ADP/ATP translocase 2	ADT2_HUMAN	33 kDa	6	
Zinc-alpha-2-glycoprotein	ZA2G_HUMAN	34 kDa	6	*
Vesicle-associated membrane protein 8	VAMP8_HUMAN	11 kDa	6	
Regenerating islet-derived protein 3-alpha	REG3A_HUMAN	19 kDa	6	*
Syncollin	SYCN_HUMAN	14 kDa	6	
Delta-1-pyrroline-5-carboxylate synthase	P5CS_HUMAN	87 kDa	5	
Cystatin-C	CYTC_HUMAN	16 kDa	5	
Elongation factor 1-alpha 1	EF1A1_HUMAN	50 kDa	4	
Protein disulfide-isomerase	PDIA1_HUMAN	57 kDa	4	
Myeloperoxidase	PERM_HUMAN	84 kDa	4	
Elongation factor 2	EF2_HUMAN	95 kDa	4	
Hemoglobin subunit beta	HBB_HUMAN	16 kDa	4	*
Transketolase	TKT_HUMAN	68 kDa	4	
Golgi apparatus protein 1	GSLG1_HUMAN	135 kDa	4	
Heat shock protein HSP 90-beta	HS90B_HUMAN	83 kDa	4	
Aminopeptidase N	AMPN_HUMAN	110 kDa	3	
Ras-related protein Rab-3D	RAB3D_HUMAN	25 kDa	3	

Actin-related protein 3	ARP3_HUMAN	47 kDa	3	
Cofilin-1	COF1_HUMAN	19 kDa	3	
Carnitine O-acetyltransferase	CACP_HUMAN	71 kDa	3	
Importin subunit beta-1	IMB1_HUMAN	97 kDa	3	
Calnexin	CALX_HUMAN	68 kDa	3	
Cadherin-1	CADH1_HUMAN	97 kDa	3	
POTE ankyrin domain family member E	POTEE_HUMAN	121 kDa	3	
Inactive gamma-glutamyltranspeptidase 2	GGT2_HUMAN	62 kDa	3	
U2 snRNP-associated SURP motif-containing protein	SR140_HUMAN	118 kDa	3	
Glyceraldehyde-3-phosphate dehydrogenase	G3P_HUMAN	36 kDa	3	*
Retinoic acid receptor responder protein 2	RARR2_HUMAN	19 kDa	3	
T-complex protein 1 subunit eta	TCPH_HUMAN	59 kDa	3	
L-lactate dehydrogenase B chain	LDHB_HUMAN	37 kDa	3	
Adenylyl cyclase-associated protein 1	CAP1_HUMAN	52 kDa	3	
Kallistatin	KAIN_HUMAN	49 kDa	2	
T-complex protein 1 subunit zeta	TCPZ_HUMAN	58 kDa	2	
Transitional endoplasmic reticulum ATPase	TERA_HUMAN	89 kDa	2	
Lysosome-associated membrane glycoprotein 1	LAMP1_HUMAN	45 kDa	2	
Serine/threonine-protein phosphatase 2A 65 kDa regulatory subunit [illegible]	2AAA_HUMAN	65 kDa	2	
Insulin-like growth factor-binding protein 2	IBP2_HUMAN	35 kDa	2	
Importin-5	IPO5_HUMAN	124 kDa	2	
14-3-3 protein zeta/delta	1433Z_HUMAN	28 kDa	2	
Catalase	CATA_HUMAN	60 kDa	2	
X-ray repair cross-complementing protein 6	XRCC6_HUMAN	70 kDa	2	
Ras-related protein Rab-27A	RB27A_HUMAN	25 kDa	2	
ATPase family AAA domain-containing protein 3A	ATD3A_HUMAN	71 kDa	2	
NADH dehydrogenase [ubiquinone] iron-sulfur protein 5	NDUS5_HUMAN	13 kDa	2	
Ras-related protein Rab-10	RAB10_HUMAN	23 kDa	2	
60S acidic ribosomal protein P1	RLA1_HUMAN	12 kDa	2	
40S ribosomal protein S10	RS10_HUMAN	19 kDa	2	
40S ribosomal protein S5	RS5_HUMAN	23 kDa	2	
Vesicle-associated membrane protein 2	VAMP2_HUMAN	13 kDa	2	
Fibrinogen-like protein 1	FGL1_HUMAN	36 kDa	2	
Heterogeneous nuclear ribonucleoprotein A1	ROA1_HUMAN	39 kDa	2	

Dipeptidase 1	DPEP1_HUMAN	46 kDa	2	
Endoplasmin	ENPL_HUMAN	92 kDa	2	
Receptor-type tyrosine-protein phosphatase F	PTPRF_HUMAN	213 kDa	2	
Beta-1,3-N-acetylglucosaminyltransferase lunatic fringe	LFNG_HUMAN	42 kDa	2	
Nucleolin	NUCL_HUMAN	77 kDa	2	
X-ray repair cross-complementing protein 5	XRCC5_HUMAN	83 kDa	2	
Cardiomyopathy-associated protein 5	CMYA5_HUMAN	449 kDa	2	
Prohibitin-2	PHB2_HUMAN	33 kDa	2	
Coatomer subunit beta	COPB_HUMAN	107 kDa	2	
Voltage-dependent anion-selective channel protein 1	VDAC1_HUMAN	31 kDa	2	
Protein disulfide-isomerase A3	PDIA3_HUMAN	57 kDa	2	
Glutaredoxin-3	GLRX3_HUMAN	37 kDa	2	
Heterogeneous nuclear ribonucleoprotein K	HNRPK_HUMAN	51 kDa	2	
Glycine--tRNA ligase	SYG_HUMAN	83 kDa	2	
Heat shock-related 70 kDa protein 2	HSP72_HUMAN	70 kDa	2	
Cell division control protein 42 homolog	CDC42_HUMAN	21 kDa	2	
Activator of 90 kDa heat shock protein ATPase homolog 1	AHSA1_HUMAN	38 kDa	2	
Annexin A5	ANXA5_HUMAN	36 kDa	2	*
DNA-(apurinic or apyrimidinic site) lyase	APEX1_HUMAN	36 kDa	2	
Apoptosis-associated speck-like protein containing a CARD	ASC_HUMAN	22 kDa	2	
BH3-interacting domain death agonist	BID_HUMAN	22 kDa	2	
Calpain small subunit 1	CPNS1_HUMAN	28 kDa	2	
Cytochrome b561	CY561_HUMAN	28 kDa	2	
Probable C-mannosltransferase DPY19L1	D19L1_HUMAN	77 kDa	2	
D-dopachrome decarboxylase-like protein	DDTL_HUMAN	14 kDa	2	
ATP-dependent RNA helicase DDX3X	DDX3X_HUMAN	73 kDa	2	
Delta(3,5)-Delta(2,4)-dienoyl-CoA isomerase, mitochondrial	ECH1_HUMAN	36 kDa	2	
Enoyl-CoA hydratase, mitochondrial	ECHM_HUMAN	31 kDa	2	
Eukaryotic translation initiation factor 3 subunit F	EIF3F_HUMAN	38 kDa	2	
Glucose-6-phosphate isomerase	G6PI_HUMAN	63 kDa	2	
Rho GDP-dissociation inhibitor 2	GDIR2_HUMAN	23 kDa	2	
Histone H2B type 1-B	H2B1B_HUMAN	14 kDa	2	
Protein HID1	HID1_HUMAN	89 kDa	2	
Isocitrate dehydrogenase [NAD] subunit beta, mitochondrial	IDH3B_HUMAN	42 kDa	2	

Ig gamma-1 chain C region	IGHG1_HUMAN	36 kDa	2
Inorganic pyrophosphatase	IPYR_HUMAN	33 kDa	2
Keratinocyte proline-rich protein	KPRP_HUMAN	64 kDa	2
L-lactate dehydrogenase A chain	LDHA_HUMAN	37 kDa	2
Lamin-B1	LMNB1_HUMAN	66 kDa	2
Lysozyme C	LYSC_HUMAN	17 kDa	2
Mitochondrial 2-oxoglutarate/malate carrier protein	M2OM_HUMAN	34 kDa	2
DNA replication licensing factor MCM7	MCM7_HUMAN	81 kDa	2
NADH dehydrogenase [ubiquinone] 1 beta subcomplex subunit 4	NDUB4_HUMAN	15 kDa	2
NADH-ubiquinone oxidoreductase chain 3	NU3M_HUMAN	13 kDa	2
Nuclear pore complex protein Nup85	NUP85_HUMAN	75 kDa	2
Obg-like ATPase 1	OLA1_HUMAN	45 kDa	2
Programmed cell death 6-interacting protein	PDC6I_HUMAN	96 kDa	2
Procollagen-lysine,2-oxoglutarate 5-dioxygenase 3	PLOD3_HUMAN	85 kDa	2
Polypyrimidine tract-binding protein 1	PTBP1_HUMAN	57 kDa	2
Ras-related C3 botulinum toxin substrate 1	RAC1_HUMAN	21 kDa	2
60S ribosomal protein L7	RL7_HUMAN	29 kDa	2
Dolichyl-diphosphooligosaccharide--protein glycosyltransferase subu	RPN1_HUMAN	69 kDa	2
40S ribosomal protein S7	RS7_HUMAN	22 kDa	2
U1 small nuclear ribonucleoprotein 70 kDa	RU17_HUMAN	52 kDa	2
Sialate O-acetylesterase	SIAE_HUMAN	58 kDa	2
Structural maintenance of chromosomes protein 5	SMC5_HUMAN	129 kDa	2
Superoxide dismutase [Mn], mitochondrial	SODM_HUMAN	25 kDa	2
Pre-mRNA-splicing factor SYF2	SYF2_HUMAN	29 kDa	2
Phenylalanine--tRNA ligase alpha subunit	SYFA_HUMAN	58 kDa	2
Isoleucine--tRNA ligase, mitochondrial	SYIM_HUMAN	114 kDa	2
Tubulin alpha-1B chain	TBA1B_HUMAN	50 kDa	2
T-complex protein 1 subunit alpha	TCPA_HUMAN	60 kDa	2
T-complex protein 1 subunit delta	TCPD_HUMAN	58 kDa	2
T-complex protein 1 subunit epsilon	TCPE_HUMAN	60 kDa	2
Thioredoxin domain-containing protein 5	TXND5_HUMAN	48 kDa	2
UBX domain-containing protein 1	UBXN1_HUMAN	33 kDa	2
UDP-glucose:glycoprotein glucosyltransferase 1	UGGG1_HUMAN	177 kDa	2
Up-regulated during skeletal muscle growth protein 5	USMG5_HUMAN	6 kDa	2

Exportin-2	XPO2_HUMAN	110 kDa	2	
40S ribosomal protein S4, X isoform	RS4X_HUMAN	30 kDa	2	
DNA-directed RNA polymerases I, II, and III subunit RPABC3	RPAB3_HUMAN	17 kDa	2	
Renin receptor	RENR_HUMAN	39 kDa	2	
Ras-related protein Rap-1A	RAP1A_HUMAN	21 kDa	2	
Nucleolar RNA helicase 2	DDX21_HUMAN	87 kDa	2	
Transmembrane protease serine 9	TMPS9_HUMAN	114 kDa	2	
SUN domain-containing protein 1	SUN1_HUMAN	90 kDa	2	
Nucleolar and coiled-body phosphoprotein 1	NOLC1_HUMAN	74 kDa	2	
Nucleosome assembly protein 1-like 1	NP1L1_HUMAN	45 kDa	2	
Fibronectin	FINC_HUMAN	263 kDa	2	
Zinc finger protein 300	ZN300_HUMAN	69 kDa	2	
Carnitine O-palmitoyltransferase 2, mitochondrial	CPT2_HUMAN	74 kDa	2	
COP9 signalosome complex subunit 5	CSN5_HUMAN	38 kDa	2	
Pancreatic secretory trypsin inhibitor	ISK1_HUMAN	9 kDa	2	*
60S ribosomal protein L4	RL4_HUMAN	48 kDa	2	*
Synaptotagmin-like protein 3	SYTL3_HUMAN	69 kDa	2	
ATP-binding cassette sub-family A member 10	ABCAA_HUMAN	176 kDa	2	
Neutrophil defensin 1	DEF1_HUMAN	10 kDa	2	*
